# Effectiveness of simulation-based cesarean section education on improving non-physician clinician midwife’s competency in performing cesarean section in Ethiopia: a quasi-experimental study

**DOI:** 10.1186/s12909-023-04968-w

**Published:** 2023-12-14

**Authors:** Fekadu Mazengia Alemu, Nigus Bililgn Yimer, Belete Belgu Kasegn, Belayneh Ayanaw Kassie, Ibrahim Yimer Ibrahim, Abdella Amano Abdo, Mulugeta Dile Worke

**Affiliations:** 1 Ethiopian Midwives Association, Addis Ababa, Ethiopia; 2 Department of Midwifery, College of Health Sciences, Woldia University, Weldiya, Ethiopia; 3 Department of Midwifery, College of Health Sciences, University of Gondar, Gondar, Ethiopia; 4 Department of Public Health, School of Public Health, Hawassa University, Hawassa, Ethiopia; 5 Department of Midwifery, College of Health Sciences, Debre Tabor University, Debra Tabor, Ethiopia

**Keywords:** Mamabirthie, Simulator, NPCMs, Simulation-based education, Quasi-experimental, Ethiopia

## Abstract

**Background:**

Simulation-based education enhances fundamental and clinical knowledge, procedural abilities, teamwork, and communication skills, as well as quality of care and patient safety. Due to excessive clinical loads and a lack of physicians, even classic teaching methods like bedside instruction are constrained in low-income settings. Thus, this study aimed to ascertain if simulation-based cesarean section education successfully raises non-physician clinician midwives’ competency in Ethiopia.

**Methods:**

A quasi-experimental study design triangulated with a qualitative design was implemented. Sixty Masters Clinical Midwifery students (29 intervention and 31 control) were taken in 5 universities. Three questionnaires (knowledge, confidence levels, and skills) were used. Qualitative data was also collected from 14 participants. The data were analyzed using SPSS version 25. Descriptive and inferential analyses were conducted. P < 0.05 was used for statistical significance. A difference-in-difference with a 95% confidence level was employed to control the potential confounders for knowledge and self-confidence. Multiple linear regression was fitted to identify the independent effect of simulation-based education interventions while controlling for other variables. Thematic analysis was performed using MAXQDA 2020.

**Result:**

The age of the respondents varies from 24 to 34 years, with the control group’s mean age being 28.8 (± 2.3) years and the intervention group’s mean age being 27.2 (± 2.01) years. The intervention and control groups’ pre-intervention and post-intervention knowledge scores showed a statistically significant difference. There was a substantial increase in self-confidence mean scores in both the intervention and control groups and between the pre-intervention and post-intervention periods in both the intervention and control groups. Furthermore, there was a substantial improvement in cesarean section skills in the intervention group as compared to the control group (59.6 (3.3) vs. 51.5 (4.8). The qualitative findings supported these.

**Conclusions:**

The study showed that simulation-based education improved students’ procedural knowledge, self-confidence, and skills. As a result, professional care teams can create simulation-based teaching packages to help students prepare for their residency.

**Supplementary Information:**

The online version contains supplementary material available at 10.1186/s12909-023-04968-w.

## Background

The United Nations (UN) has set a Sustainable Development Goal to decrease the global maternal mortality ratio to 70 per 100,000 live births by 2030. Although Ethiopia has made notable progress toward reducing maternal and perinatal mortality rates, it is still a national public health challenge [1]. According to the 2016 Demographic and Health Survey of Ethiopia (EDHS), the national maternal mortality rate was 412 per 100,000 live births [2]. Maternal and neonatal complications are relatively higher in cesarean section deliveries [3].

Simulation-based medical education (SBE) is an educational activity that applies different simulations to replicate clinical cases [4]. Simulation-based training is becoming popular and has brought a paradigm shift in medical education by improving providers’ clinical competency [5]. Simulation has been shown to enhance basic and clinical knowledge, procedural skills, teamwork, and communication skills, including patient safety and quality [6]. It is a relatively new educational method in low-income countries [7]. Even traditional teaching methods, such as bedside instruction, are limited in low-income settings due to overwhelming clinical volumes and physician shortages [7].

Cesarean section (CS) is the most performed procedure globally [8]. Simulation-based cesarean section education can provide health professionals with a safe and controlled environment [8, 9]. Unlike no training, obstetric simulation-based training improved specific maternal and perinatal outcomes [10]. In low- and middle-income settings, simulation-based training is practical in knowledge retention and skills [11, 12]. Similarly, simulation-based education has shown better self-efficacy and knowledge retention in obstetric resident training in low and middle-income countries (LMICs) [13]. A reduction in the incidence of postpartum hemorrhage by 38% was reported after the introduction of obstetric simulation-based training in a low-income setting [14].

Inadequate knowledge and experience on cesarean section lead to increased surgical errors and unfavorable patient safety [15]. Surgical mistakes involving blood vessels and the bladder are common during cesarean delivery [16]. On the contrary, a retrospective finding from Nigeria showed the surgeon’s skill was not a significant factor in high maternal deaths [17].

Establishing simulation centers in Rwanda and Ethiopian teaching hospitals demonstrated positive attitudes, high utilization, and sustained knowledge transfer [18, 19]. The associate clinician postgraduate surgical task-sharing training program in Sierra Leone considerably reduced overall operation time among trainees [20].

In Ethiopia, Clinical Midwifery is one of the non-physician postgraduate training programs with comprehensive emergency obstetric care (CEMOC) competencies, including cesarean section. Studies showed non-physician clinicians (NPCs) performed a significant proportion of CEMOC and were as competent as physicians in reducing maternal and fetal deaths [21]. Education and training for NPCs, including Clinical Midwives, would be key strategies to improve maternal and child health [22, 23].

Implementing simulation-based training has been challenged by infrastructural limitations, including equipment, inadequate time for exercise, the higher number of participants, and sustainability issues after the training [24]. Qualitative evidence showed the practicality of simulators was limited by institutional and staff turnover factors in rural Alaska [25]. Furthermore, building simulation centers with high-fidelity manikins might not be cost-effective in developing countries [26].

Laerdal Global Health created the MamaBirthie Cesarean Section (CS) to help physicians prepare for simple and complex births. MamaBirthie and a module for training cesarean sections are built into the simulator. These skills include abdominal/uterine opening, cephalic/breech birth, delivery of a deeply engaged head, severe postpartum hemorrhage through B-Lynch suture, and suturing of abdominal and uterine layers. The simulator can be reused for 1500 delivery cycles and suturing [27]. Evidence about cesarean section simulators’ educational and clinical effectiveness is limited [28].

We hypothesized that employing the Mamabirthie CS for simulation-based cesarean section training would increase learners’ competency. Therefore, the current quasi-experimental study aimed to determine how well NPC midwives’ competency was increased by simulation-based cesarean section teaching utilizing Mamabirthie CS. Additionally, we evaluated the NPC Midwives’ and their teachers’ experiences with simulation-based instruction.

## Methods

### Study setting

The study was conducted in 5 public Universities providing MSc in clinical Midwifery Specialist training to produce Non-Physician Clinician Midwives who can perform a cesarean section. During the commencement of the study, seven universities were providing the training program in Ethiopia (University of Gondar, Hawassa University, Wollo University, Arbaminch University, Debre Tabor University, Bahir Dar University, and Mekelle Universities). The study included five Universities out of the seven; two universities were excluded (Mekelle University was not operating because of conflict situations, and Bahir Dar University was excluded because it didn’t accept students for the respective academic year). There was an average of 7–8 students in each University.

The University of Gondar has 45,000 students in 144 programs. The College of Medicine and Health Science has 28 master’s programs in medicine, public health, biomedical science, nursing, and midwifery. Midwifery training at Gondar College of Medicine and Health Sciences is among the earliest midwifery training in Ethiopia. In 1998, the direct diploma was awarded; in 2004, the first post-basic BSc was awarded; and in 2005, the first generic bachelor midwifery training was begun. In 2010, the Department of Midwifery expanded the program to Masters in Clinical Midwifery in the country for the first time. Then, in 2017/18, the department continued its breakthrough by launching a PhD program in Midwifery and Women’s Health, the first in East Africa. In 2018, the Department of Midwifery then upgraded to the school level named “School of Midwifery,” which contains three departments (the Department of General Midwifery, the Department of Clinical Midwifery, and the Department of Women’s and Family Health). The School of Midwifery at the University of Gondar is by far the largest midwifery training institute in Ethiopia, providing midwifery training both at the undergraduate and postgraduate levels.

Arba Minch University (AMU) is located in Arba Minch town, which is located 505 km southwest of Addis Ababa, the capital of Ethiopia. Arba Minch University is a National Research University with about 36,000 students enrolled in first, second, and third-year regular, evening, weekend, and distance learning courses. The University has over 73,000 graduates in its 35-year journey of PhD 14, MSc/MA 6,270, BA/BSc 64,800, Diploma and advanced diploma 1896. Currently, Arba Minch University runs 34 PhDs, 140 Master’s, and 76 Undergraduate programs. Arba Minch University launched its midwifery program in 2010 as part of the College of Medicine and Health Sciences, and the department reached a milestone in 2017 when it expanded the program to offer master’s level training in clinical midwifery. From 2017 to date, 52 graduates have completed the Clinical Midwifery program. Additionally, the program teaches 12 graduate candidates and 14 additional first-year students.

### Study design

A mixed methods study was conducted between April 1, 2022, and March 30, 2023. A quasi-experimental study was conducted with a pre-test and post-test design to evaluate the effectiveness of simulation-based education on cesarean section competency of non-physician clinician midwives (NPCMs). Then, a post-intervention phenomenological qualitative study was conducted to explore the experiences of simulation-based education among intervention groups. The quantitative study investigated the effectiveness of simulation-based cesarean section education on NPCM’s knowledge, confidence, and skills. In contrast, the phenomenological study examined the experiences of simulation-based cesarean section education among students in the intervention group who had not participated in the quantitative research.

### Study participants

The study included 62 NPC midwives (2nd year MSc in clinical Midwifery Specialist) students (29 intervention and 33 control) from 5 Universities. Those 2nd years, MSc in clinical midwifery students who completed all theoretical courses and were ready to start clinical practice were included. Study participants were assigned proportionally to the University’s MSc clinical midwives’ enrollment capacity. The five universities were categorized using the lottery method as intervention group (the University of Gondar and Arba Minch University) and control group (Wollo University, Debre Tabor, and Hawassa Universities). The number of students assigned to each University were: University of Gondar (20 students), Arbaminch University (9 students), Wollo University (12 students), Hawassa University (9 students), and Debre Tabor University (10 students).

On the other hand, the 14 participants in the qualitative study were eight students, three gynecologists and obstetricians, and three midwifery department heads. These participants had been enrolled in simulation-based education for four months.

### Intervention

The intervention is a simulation-based cesarean section education program using the MamaBirthie CS simulator. MamaBirthie CS is a birthing and safe c-section simulator that allows trainees to prepare for cesarean sections and other delivery problems. According to Laerdal’s practical approach to training with MamaBirthie CS, trainees display and practice evidence-based surgical methods during cesarean section (*MamaBirthie CS - Birthing and safe c-section simulator | Laerdal Global Health*). Suturing the uterine and abdominal layers and delivering the baby and placenta are among the methods used. It is made of long-lasting textile materials, allowing the simulator to be reused for 1500 delivery cycles and 500 cesarean deliveries with sutures without extra consumables like lubricants or replacements. A facilitator or peer can wear the simulator during skills training, encouraging learners to integrate polite conversation with the patient in their activity (Supplementary Fig. 1).

To implement the intervention, the first MamaBirthie CS simulator, Cesarean section set, operation room supplies, and personal protective equipment were provided for each intervention university to ensure the simulation center mimics the operation room setup. Then, the simulation-based cesarean section education facilitation and MamaBirthie CS simulator training were provided for obstetricians who delivered the simulation intervention using Faculty Guide: A Practical Guide to Training with MamaBirthie CS. At the commencement of the intervention, a brief orientation on using the Mamabirthie CS simulation simulator was also offered to students in the intervention group.

The simulation training was delivered by an experienced senior obstetrician who got a simulation-based training instructor course. The instructor first demonstrates the cesarean section procedure in each session with a whole part-whole approach. Cesarean section procedure learning guides were used. The students then demonstrate and practice cesarean delivery, scrubbing, skin incision, fascia entry, peritoneum separation, uterine incision, fetal extraction, placenta delivery, and layer-by-layer closure. Each session was demonstrated with discussions and facilitator lead group debriefing. While the students demonstrate, the instructor observes and provides feedback on each step of the procedure using a checklist containing a summary of the key actions in the scenario. The simulation-based education was provided for 4 h a week and continued for three months, making 12 simulation-based education sessions. The focus of each session was well outlined, from the simple and broken steps to a complex and complete procedure performance.

### The control groups

The students in the control group didn’t get any intervention. They attended the routine academic practice (morning sessions, bedside teaching, rounds, and seminars).

### The outcome

The primary outcome was cesarean section skill competence of Clinical Midwifery students in performing CS. The secondary outcomes were knowledge of cesarean section and self-confidence in doing cesarean section among participants.

### Sample size and sampling procedure

We took all 2nd year MSc in clinical Midwifery students in 5 universities providing the program in the country (29 intervention and 31 control). A power calculation demonstrated that, with a sample size of 60, the study has a power of > 90% to detect an effect size of Cohens’d 1.96 in CS Objective structured assessment of technical skill (OSATS) scores (comparing OSATS scores in intervention and control groups) at a significance level of 0.05 assuming the independent sample t-test (Supplementary Fig. 2).

Purposive sampling was used to choose NPCMs, Midwifery Department Heads, Obstetricians, and Gynecologists (OBGYNs) from several intervention universities. The 14 interviews came from the University of Gondar (two OBGYNs, five students, and two department heads) and Arba Minch University (one OBGYN, one department head, and three students). The study participants were enrolled indefinitely until the data were saturated.

### Research instrument

The data collection tool includes socio-demographic information, knowledge assessment questions, cesarean section performance self-confidence assessment questions, OSATS skill assessment, and the task-specific cesarean section skill global rating scale. Participants’ knowledge and self-confidence were assessed during and post-intervention periods for both groups using self-administered questions.

The objective structured assessment of technical skill (OSATS) tools developed by the Royal College of Obstetrics and Gynecology and other literature [29] was used to assess the skill competency of students in performing CS for both intervention and control groups. A 15-item Likert scale self-confidence assessment checklist adapted from Geoffrion R et al. [30] was used to assess the students’ self-confidence in performing the cesarean section procedure. The OSATS global checklist has 13 items and is measured using a Likert scale from 1 to 5 (1 unable to perform the task, 5 = perform the task easily) for each item (Cronbach’s Alpha = 86%). The task-specific global rating scale for CS with 13 items Likert scale question rating from 1 to 5 (1 = unable to perform the task, 5 = efficiently) with Cronbach’s Alpha = 92%. A 15-item Likert scale self-confidence assessment checklist adapted from Geoffrion R et al. [30] assessed the students’ self-confidence in performing the cesarean section procedure.

As the students in both the intervention and control groups had never been exposed to CS skills, we didn’t assess the baseline skill competence for both groups. The skills of both intervention and control were evaluated using OSATS at the end of the intervention. The knowledge and self-confidence were assessed during the baseline and at the end of the intervention for both groups. For the qualitative study, interview guides were prepared separately for MSc Midwifery students, Instructor obstetricians and gynecologists, and heads of departments. The guides were designed in English, translated into Amharic, and back-translated to English to check consistency. All interviews were taped, and field notes were obtained.

In-depth interviews were conducted by four researchers, one facilitating the conversation and the other arranging the places, communicating with candidate interviewees, and taking notes as needed. All interviews were audio-recorded with the participant’s permission. Each interview lasted 16 to 37 min, with an average of 25 min. Participants were served tea, coffee, water, and soft drinks to thank them for their time.

### Data quality assurance

Senior obstetricians and gynecologists did data collection for the cesarean section skill assessment. Tools were developed with simple and easily understandable English. The Pre-test was done among obstetric residents. Then, appropriate language, context, and instruction customizations and question sequence and word modifications were made to the tools.

Before the commencement of data collection, orientation, idea exchange, and demonstration training were provided for data collectors and supervisors. The data collection process was closely supervised. Questionnaires were checked for completeness, and immediate actions were taken for incomplete data.

The obstetrician and gynecologist who assessed students’ skills were unaware of the participant group allocation or the evaluation time point (pre- or post-intervention). The assessment was not a part of the simulation-based education intervention.

Concerning the study’s trustworthiness, the investigators implemented several strategies to enhance credibility, transferability, dependability, and conformability. Information was provided clearly and exhaustively to the study participants to build a trustful relationship. During the interpretation of the quantitative findings, detailed views of the meaning of the phenomenon or concept for individuals were considered.

### Data management and analysis

After entering the data, it was exported to the SPSS version 25 software package and Excel for data validation, cleaning, and analysis. It was then transferred to STATA 15 for an additional examination. Cleaning was achieved using frequency computation and sorting. Mean scores were utilized to describe the CS performance of Master of Science Clinical Midwifery students in both the intervention and control groups. The participants’ pre- and post-knowledge of CS and self-confidence were assessed using a paired t-test. After validating the homogeneity of variance assumption, an independent sample t-test was employed to observe the statistical difference in mean competency scores between intervention and control groups. P-values of less than 0.05 were used to determine statistical significance. A difference-in-difference (DID) test with a 95% confidence level was used to compensate for potential confounders in knowledge and self-confidence. The independent effect of SBE intervention was identified using multiple linear regression while controlling for other variables.

The recorded audios were transcribed and then translated into English by an expert proficient in both languages for the qualitative data, which was then analyzed using the thematic analysis approach with MAXQDA 2020. Before coding, the sentences were cross-checked with audio files for accuracy and consistency. A research assistant, a university graduate with experience doing qualitative research and preparation for information aggregation, produced the copies. Experts in qualitative data analysis read a sub-sample of transcripts to ensure consistency. The data was analyzed using a theme analysis approach. We read and reread the descriptive material to become acquainted with the data and obtain theme analysis codes. The analysis method integrated prior codes with data-driven codes based on the research topic. An open coding method was adopted to execute information-driven codes and categorize small codes. The little codes were organized into significant themes, and emergent issues were used as analytical categories. The codes were then categorized into potential broader subthemes/themes, and associated data was collected within each prospective subtheme/theme. The structure of all coded data extracts was examined under each subtheme/theme. Those that formed a regular pattern within subthemes/themes were investigated. As a result, an analytical thematic map was created. In 12 subthemes, the essence of each subtheme/theme was found and explained to readers. The subthemes were categorized based on their content, and these subthemes were combined to form three themes. Participant quotations were italicized in the text with a unique identification number showing the participant code, age, and sex to support the emerging themes and subthemes.

## Results

### Participant characteristics

Sixty people (29 intervention and 31 control) were enrolled in the trial. The age varies from 24 to 34 years, with the control group’s mean age being 28.8 (± 2.3) years and the intervention group’s mean age being 27.2 (± 2.01) years. There were 12 (41.4%) and 9 (29%) females in the intervention and control groups. In the intervention and control arms, 9 (31%) and 10 (32.3%) participants work in healthcare facilities, respectively. Half (51.7%) of the intervention group and 42% of individuals in the control group had 1–4 years of clinical experience before enrolling in the program. Only 5 (17.2%) intervention group members had simulation-based learning experiences before the intervention. There is no statistically significant difference in respondent characteristics between the intervention and control groups (Supplementary Table 1).

On the other hand, eight master’s clinical midwife students and three obstetricians and gynecologists were among the fourteen participants in the qualitative study. The participants’ ages ranged from 30 to 40 (Supplementary Table 2).

### Knowledge of cesarean section procedure among participants

No significant knowledge score difference exists between the intervention and control groups during the baseline (*P = 0.12*) and post-intervention periods (*P = 0.08*). However, there is a statistically significant difference between pre-and post-intervention knowledge scores within the intervention (*P = 0.001*) and control groups (*P = 0.001*). A difference-in-difference analysis was performed to determine the average intervention effect, and no significant difference was found between the two groups (DID = 0.5, P = 0.90) (Table 1).

**Table 1:** Knowledge of cesarean section among participants for simulation-based education to improve non-physician clinician midwives’ cesarean section competence in Ethiopia, 2023

Assessment	Control (C)	Intervention (I)	Difference (I-C)	P-value
Baseline (B)	48.4	53.3	4.9(−1.4, 11.2)	0.12
End line (E)	63.9	69.3	5.4(−0.7, 11.6)	0.08
Mean difference (E-B)	15.5(10.2, 20.8)	16(10.1, 22.0)		
P-value	< 0.001	< 0.001		

The participants in the qualitative study explained that the simulation-based education helped them fill their knowledge gaps, familiarizing them with the layers of the abdomen, the procedure, and the operation room environment and retaining their knowledge:

…, my understanding of the anatomical layers, the steps of the techniques, and the incision types have improved, and I am now working on steps with justification. It also familiarized me with the equipment, the layers of the body, and the operating room environment. Further, it is one of the best teaching techniques which creates opportunities to increase my knowledge retention. (a 24-year female student)

Another participant added:

Simulation-based teaching allows us to acquire knowledge that we can use in actual operation room actual circumstances. (a twenty-five-year male student)

Their trainer also added:

It simulates actual patients, which helps students understand the anatomical structure of the actual patient’s abdomen and uterus. (32 years old male OBGYN with nine years of experience)

### Self-confidence towards performing cesarean section among participants

The confidence level regarding cesarean section procedures during the pre-test showed no significant difference between intervention and control groups (P = 0.30) at baseline. However, there was substantial self-confidence score improvement between the intervention and control groups during the post-intervention period (P ≤ 0.001). Likewise, there was a significant difference in self-confidence score improvement between pre-and post-intervention in the intervention (mean = 20.6 (17.0,24.2), *P = 0.001*) and control groups ((mean = 15.7 (11.2,20.2), *P = 0.001*). However, the difference in difference analysis does not show a significant difference between both groups (*DID = 4.9, P = 0.25*) (Table 2).

**Table 2:** Self-confidence towards performing cesarean section among participants for simulation-based education to improve non-physician clinician midwives’ cesarean section competence in Ethiopia, 2023

Assessment	Control (C)	Intervention (I)	Difference (I-C)	p-value
Baseline (B)	47.3	51.2	3.9(−3.7, 11.5)	0.30
End line (E)	63	71.8	8.8(5.1, 12.4)	< 0.001
Mean difference (E-B)	15.7(11.2, 20.2)	20.6(17.0, 24.2)		
	< 0.001	< 0.001		

The simulation-based teaching also improved the perceptions of the operating room staff, boosted their confidence, reduced students’ anxiety, and accelerated their engagement in conducting actual procedures, according to the participants in the qualitative study.

…the students who signed up after the simulator was installed were eager to perform a cesarean section and started the procedure 6 to 7 months before the practice period ended. They are asking us to perform CS, and they are becoming more assured. (35 years old male OBGYN with eight years of experience)

The practice on the simulator was very helpful for us. […]. We were expected to observe procedures in the operation room simply. However, the simulation-based education allowed us to demonstrate the techniques earlier, which boosted our confidence and made our actual practice smooth. (a twenty-eight-year-old male student)

Another Obstetrics and gynecology specialist added:

…Simulation-based training helped to dispel anesthetists’ and scrub nurses’ misconceptions that students will spend long hours conducting procedures. Another significant advantage of simulation-based learning was the improvement of communication between OR personnel and students. Before the actual treatments, simulation-based instruction proved crucial in lowering fear and boosting confidence. (32 years old male OBGYN with nine years of experience)

They also observed that the simulation-based instruction helped them minimize their time assistance, promote peer learning, speed up the operation, and enhance their willingness to do cesarean sections.

Well! Let us start with self-assurance. […], students who engaged in activities such as suturing practice on the simulator would be fine with conducting basic procedures in the operation theatre. As a result, the surgeons had more confidence in the student and needed to observe him for less time. (a twenty-five-year-old male student)

Another student added:

As for one’s self-confidence, it increases by more than 100% because we talk about it with our peers. This is because when the senior staff took the trainees to the hospitals, the trainees only had the opportunity to collaborate independently with the teacher’s supervision. However, when we work in groups with the senior staff, we have many opportunities to share experiences based on the observations of the teachers and our peers. We gained knowledge of several crucial ideas and observed the newest program tools, allowing me to conclude that the program is efficient. (a 25-year-old female student)

### Cesarean section competence among participants

**Fig. 1:**
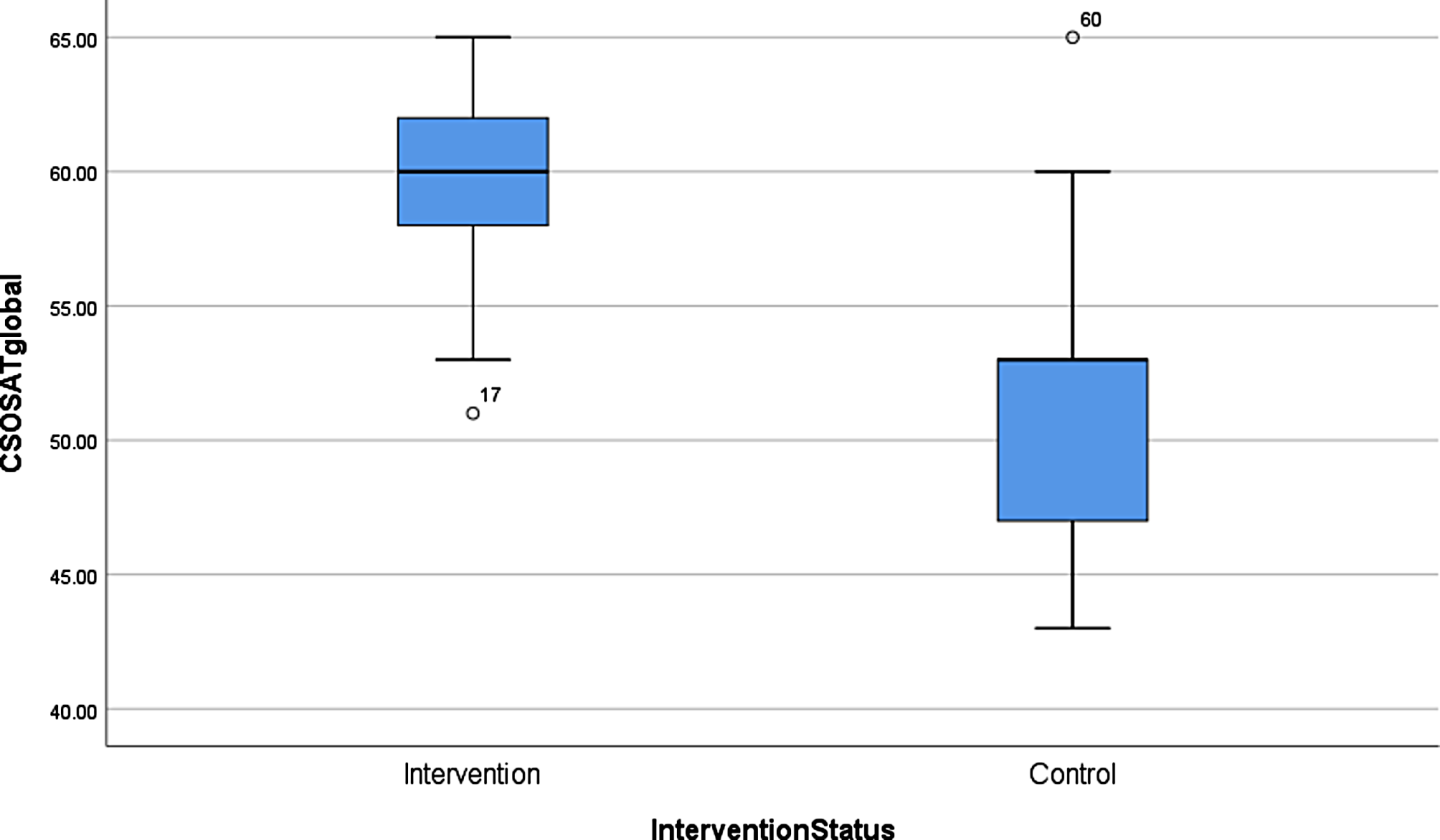
Box plots with objective structured assessment of technical skills (OSATS) scores in intervention and control groups

All study participants demonstrated enhanced skills in the cesarean section. OSATS scores were significantly higher in the intervention group compared to the control group, 59.6 (± 3.3) vs. 51.5 (± 4.8), respectively; p < 0.001). Figure 1 shows a box plot of OSATS scores in both groups, indicating that simulation-based cesarean section education improves the participants’ skills in performing a cesarean section (Fig. 1).

**Fig. 2:**
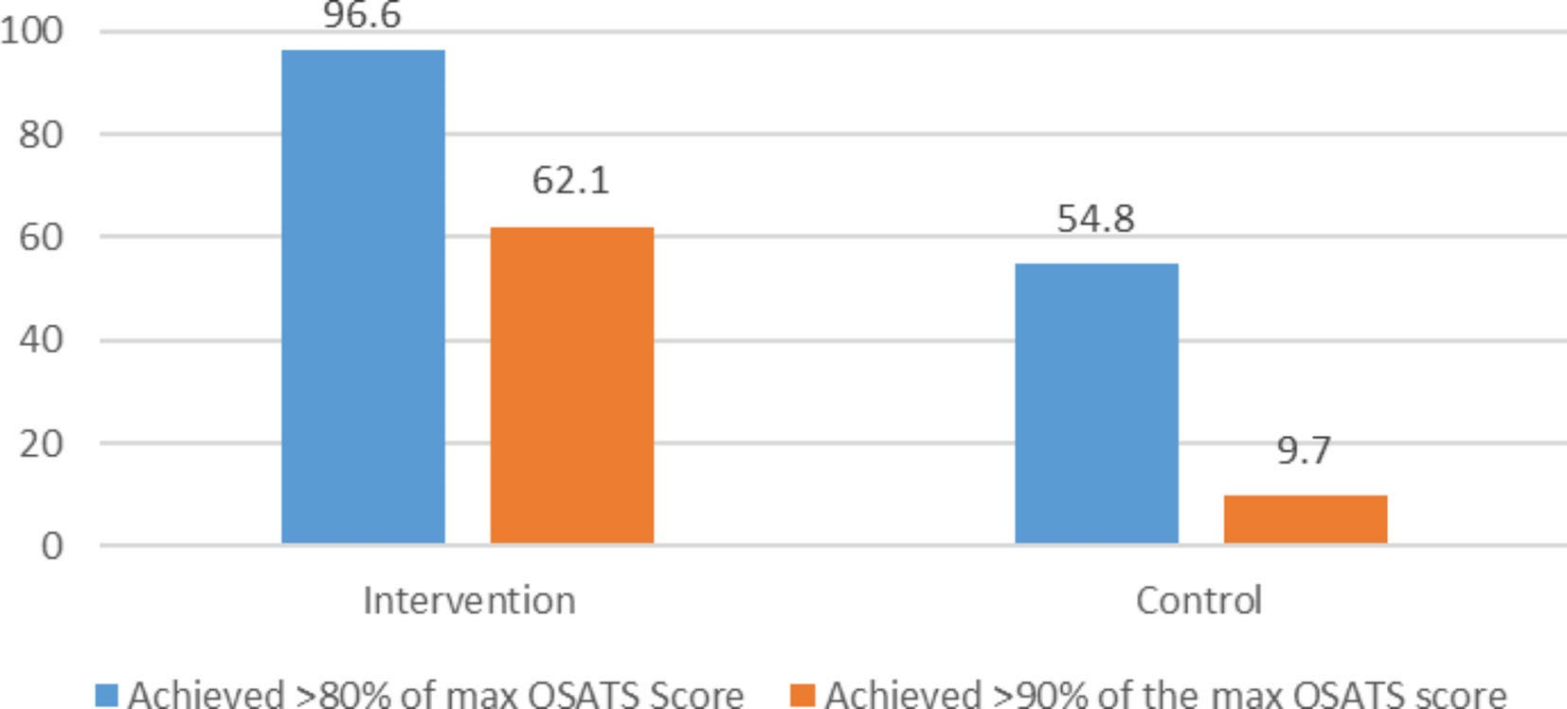
Number of participants achieving > 90%, > 80% of the maximum CS OSATS scores during the intervention and control groups, 2023

The number of subjects achieving 90% and 80% of the maximum OSATS scores in the intervention and control group were 18/29 (62.1%), 28/29 (96.6%), and 3/31 (9.7%), 17/31 (54.8%) (Fig. 2).

The task-specific cesarean section performance global rating (GR) also showed a significant difference between the intervention groups 62.5 (± 2.1) and control groups 52.2 (± 4.3); p < 0.001), favoring the intervention group (Table 3).

**Table 3:** Skill competence among participants for simulation-based education to improve non-physician clinician midwives’ cesarean section competence in Ethiopia, 2023

	Control (n = 31)	Intervention (n = 29)	Mean score Difference	t-value	P-value
OSATS scores	51.5 (± 4.8)	59.6(± 3.3)	8.1(6.0,10.3)	7.6	< 0.001
Task-specific performance global rating scale	52.2(± 4.3)	62.5(± 2.1)	10.4(8.6,12.1)	11.7	< 0.001

To control for possible confounders, we analyzed multiple linear regression by including experience in simulation-based education, sex, workplace, clinical expertise, ever-assisted CS, knowledge, and self-confidence. After controlling for those variables, the simulation-based cesarean section education intervention significantly improved the cesarean skill competence of NCPMs (β = 703, 95% CI 4.35, 8.71). Finally, we found that there is no association between sex, workplace, clinical experience, ever-assisted CS, knowledge, and self-confidence (and cesarean section competence among NPCMs at P-value = 0.05 (Table 4).

**Table 4:** Multivariable linear regression of factors associated with skill competence among participants for simulation-based education to improve non-physician clinician midwives’ cesarean section competence in Ethiopia, 2023

Variables		Estimate (β)	P-value	95%CI
Intervention status	Intervention	7.03	< 0.001	4.35, 9.71
	Control	Reference		
Sex	Male	1.16	0.46	−1.94, 4.25
	Female	Reference		
Clinical experience before	No clinical experience	Reference		
	1–4 years	−0.77	0.61	−3.86, 2.31
	5 years and above	0.10	0.96	−3.71, 3.92
Workplace	Higher education institution	0.39	0.81	−2.86, 3.65
	Health facility	Reference		
Marital status	Married	1.30	0.34	−1.39, 3.99
	Single			
Experience on SBE	Yes	1.04	0.47	−1.83, 3.91
	No	Reference		
Ever assisted CS	Yes	Reference		
	No	1.18	0.40	−1.61, 3.96
Self-confidence to perform CS		0.15	0.08	−0.02, 0.31
Knowledge on CS		0.02	0.64	−0.08, 1.13

The qualitative findings also revealed that the students had the chance to develop their skills, such as basic surgical skills, communication skills, and decision-making skills.

The simulation-based CS practice has significantly changed me. First, it allowed me to practice knotting, handling sticks, and various operation process types and steps before the practice session. Second, it helped me become accustomed to and comfortable with actual practice. (a thirty-year-old male student)

Other students added:

The simulator, which I view as the ideal client, enabled me to control typical handling and management errors in real-world setups. Additionally, it assisted me in improving my abilities before engaging in genuine patient handling. (a 27-year-old female student)

The opportunity of getting training on the simulator helped me to recognize and correct errors before the actual practice, which was crucial for my decision-making and practical skill. (a thirty-four-year-old male student)

Other participants added:

Simulation-based education gave us another channel of connection with our seniors (Instructors) to share experiences. It made it possible to demonstrate basic surgical skills before dealing with real patients. (a twenty-five-year-old male student)

…, simulation-based education improved student communication abilities with classmates and the operating room staff. (32 years old male OBGYN with nine years of experience)

Furthermore, participants explained that simulation-based education increased participants’ confidence, knowledge, and skills, improving patient prognosis and building program image:

…, it can help with image-building, which means that other hospital experts may think that the clinical midwifery program is a significant and highly effective one when trainees enter the real workplace and carry out tasks as they should and make decisions effectively because they take part in demo classes where they practiced these tasks. Additionally, if we decide to leave our academic careers, it will enable us to get employment anywhere. (25 years old female student)

Another participant added:

Numerous elements affect client safety. These include the biological makeup of the client, nutritional status, and medical issues. The patient’s factors [biological makeup and diet] may or may not be adjustable. However, it is important to monitor the physician-related aspects properly. The most crucial element of the physician aspect is tissue handling. For a wound to heal quickly and a client to be released from the hospital quickly, tissue handling is crucial. Inadequate tissue handling would also cause re-treatment or a delay in wound healing. Thus, having the best post-operative patient prognosis would be made possible by students properly demonstrating their use of simulation-based instruction. (32 years old male OBGYN with nine years’ experience)

## Discussion

The present study scrutinized the effectiveness of simulation-based education in raising non-physician clinician midwives’ competency in Ethiopia. The results indicated no statistically significant knowledge differences among the intervention and control groups. This could be due to the routine practice, which contains bedside teaching, morning sessions, seminars, and rounds, which may increase the students’ knowledge in both groups. However, in the qualitative study, NCPMs participating in this study pointed out that simulation-based cesarean section education with MamaBirthie CS improves their knowledge of the indications for CS, anatomical layers, and procedural steps to perform a cesarean section. They also mentioned that it enabled them to familiarize themselves with the instruments and suturing. This finding agrees with an East Carolina University US study that revealed that cesarean section practice using mannequin simulation demonstrated substantial knowledge improvement, particularly in suturing ability [31]. This finding was also supported by another recent study in Indonesia, which found that simulation-based education intervention with a mannequin enhanced obstetric residents’ knowledge of cesarean section [32].

In this study, the baseline self-confidence score did not significantly differ between the two groups. However, during the post-intervention period, there was a substantial increase in the mean self-confidence score between the intervention and control groups. The mean score improvement between the pre-intervention and post-intervention self-confidence scores in both the intervention and control groups is also statistically significant. The lack of a substantial difference among the two groups, as indicated by the difference in difference analysis, suggests that the difference is not the result of the intervention. The simulator-based cesarean section education, on the other hand, according to the qualitative participants, increased their procedural confidence.

However, consistent with other findings [33], our result revealed that NPMs’ self-confidence was not improved due to simulation-based education. This finding contradicts other findings [34, 35]. The scientific community may be misled because self-confidence does not always correlate with performance ability. However, according to other studies, self-administered questionnaires with response and social desirability biases have improved self-confidence. This may be because the respondent feels undervalued due to reporting their precise level of confidence. Thus, further studies should be conducted in this regard.

The findings of this study demonstrated that simulation-based learning improves master’s clinical midwife students’ abilities to perform cesarean sections across all intervention groups. This was evident in task-specific cesarean section performance rating assessments and the objective structured assessment of technical skills (OSATS). Since simulation-based learning for cesarean sections is a tried-and-true approach to instructing medical professionals [36], it revealed improvement in students’ competency, quality of client care, and better patient prognosis. This suggests that to maximize patient outcomes, reduce risks, and enhance clinical decision-making, midwives involved in simulation-based learning of cesarean section skills must be able to meet a high clinical skills standard that was rigorously tested.

This finding is consistent with a study in India [37]. However, our study’s measurement (OSATS and task-specific) is relatively more comprehensive than the Indian study (self-perceived). The participants in our research (both interventions and controls) were observed using the mentioned checklists while performing a cesarean section on the simulator. In addition, as discussed above, the authors assessed students’ knowledge and self-perceived confidence in performing cesarean section simultaneously. Similar to a study finding in the USA [31], this study revealed that using a simulation model at the end of theoretical sessions or early in practical attachment has the most excellent positive effect on students’ Cesarean section performance skills. Thus, consistent with other findings [28, 38], simulation-based Cesarean section education has shown a proven advantage in training master clinical midwives in low-resource settings with minimal patient flow.

This study illustrated the critical contribution non-physician clinicians (NPCs) can make to settings with limited resources. First, it makes the case that the deployment of NPC midwives in settings with limited resources might help reduce the rate of maternal death, especially in places where obstetricians and doctors are in short supply, which is one of the targets of Ethiopia [39]. This might be accomplished by having quick access to trained birth attendants, emergency obstetric surgery, blood transfusions, and other life-saving measures. These measures could also improve the prognosis for newborns by preventing stillbirths and neonatal deaths through prompt and appropriate care for complications during childbirth. Second, it taught low- and middle-income countries how to reach communities that are hard to reach with mid-level comprehensive emergency obstetric care services. This can empower women by giving them control over their reproductive health and lowering the anxiety and fear of childbirth in settings with limited resources. Thirdly, it can help enhance overall health systems in resource-constrained situations, resulting in increased access to healthcare for all community members. It also lowers the costs involved with training a cesarean section using various modalities, such as the cadaver. Furthermore, non-physician clinical midwives training can help communities economically in the long run by lessening the strain on healthcare systems and boosting female output through the reduction of maternal mortality and improvement of maternal and newborn outcomes.

Though the study’s strength relies on the combination of different tools for the competency assessment, the mixed approach of the study, and the multi-institutional nature of the samples, it also had some limitations, which should motivate further research. First, the quasi-experimental design limited the study’s ability to conclude a causal association between the intervention and observed outcomes. Second, since the observation is conducted on the simulator, the lack of observation of the SBE effect on the subsequent clinical practice and client outcomes is a limitation that needs further study.

## Conclusion

Overall, simulation-based education using the Mamabirthie cesarean section simulator revealed improvement in the participants’ skills, self-confidence, and knowledge. Thus, all universities receiving students for the MSc clinical midwifery training should implement simulation-based cesarean section education before starting clinical attachment. Moreover, future studies can examine the effect of simulation-based education on subsequent clinical practice and patient outcomes.

### Electronic supplementary material

Below is the link to the electronic supplementary material.

**Supplementary Material 1**: Figure 1. Students practicing C-section on Mamabirthie simulator

**Supplementary Material 2**: Figure 2. Study participant selection process flow diagram

**Supplementary Material 3**: Table 1. Participant characteristics for simulation-based education to improve non-physician clinician midwives’ cesarean section competence in Ethiopia, 2023

**Supplementary Material 4**: Table 2. Demographics characteristics of qualitative participants for simulation-based education to improve non-physician clinician midwives’ cesarean section competence in Ethiopia, 2023

## Data Availability

All data generated or analyzed during this study and the additional files are included in this manuscript.

## Abbreviations

CEMOC: comprehensive emergency obstetric care
CS: Cesarean section
DID: Difference-in-Difference
EDHS: Demographic and Health Survey of Ethiopia
IRB: Institutional Review Board
LMICs: low and middle-income countries
NPCMs: non-physician clinician midwives
NPCs: non-physician clinicians
OBGYNs: obstetricians, and Gynecologists
OSATS: Objective structured assessment of technical skill
UN: United Nations
