# Supplementary figures and images for: Effectiveness of simulation-based cesarean section education on improving non-physician clinician midwife’s competency in performing cesarean section in Ethiopia: a quasi-experimental study
Source: BMC Med Educ. 2023 Dec 14;23:961. doi: 10.1186/s12909-023-04968-w (PMC10722683; doi:10.1186/s12909-023-04968-w)

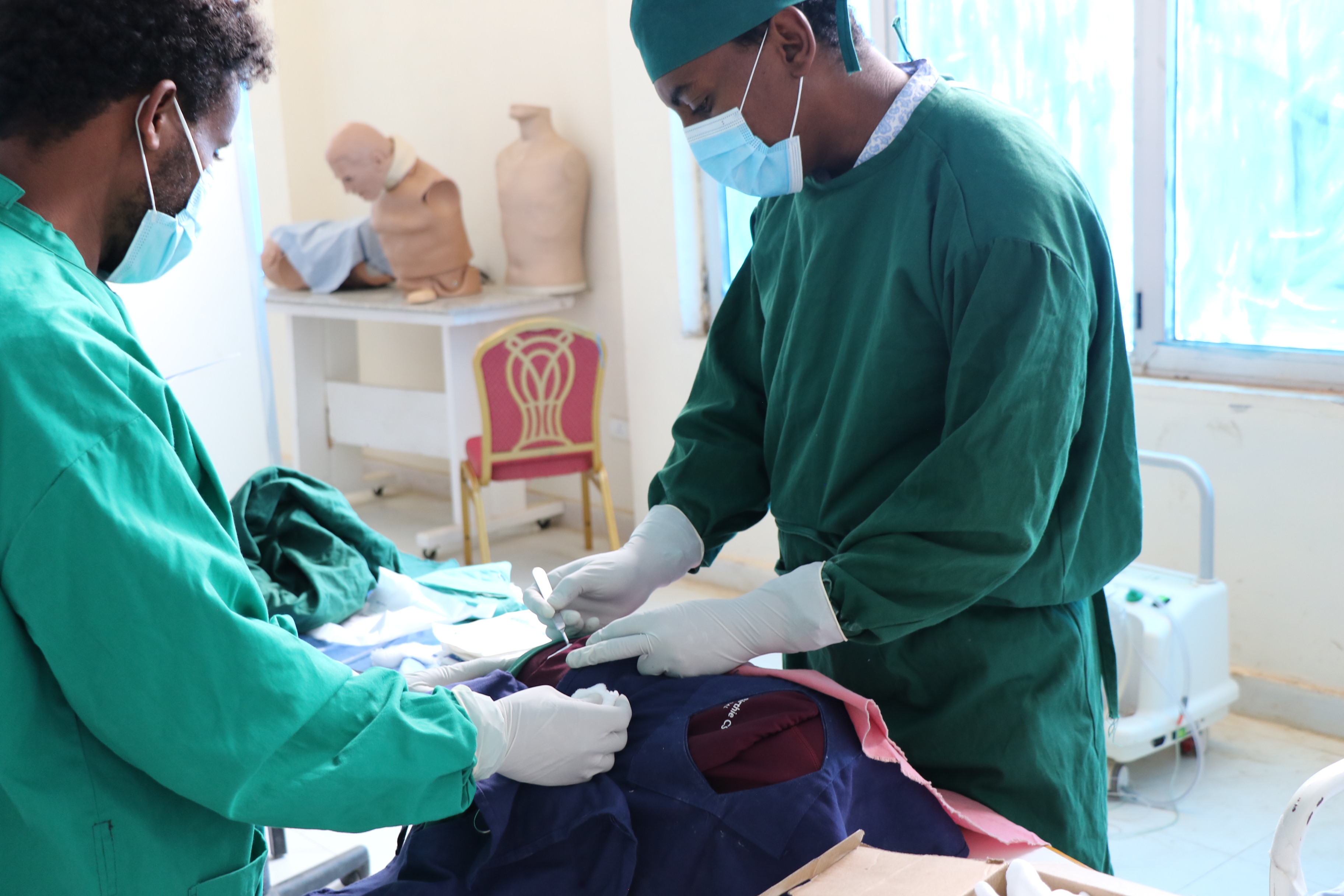

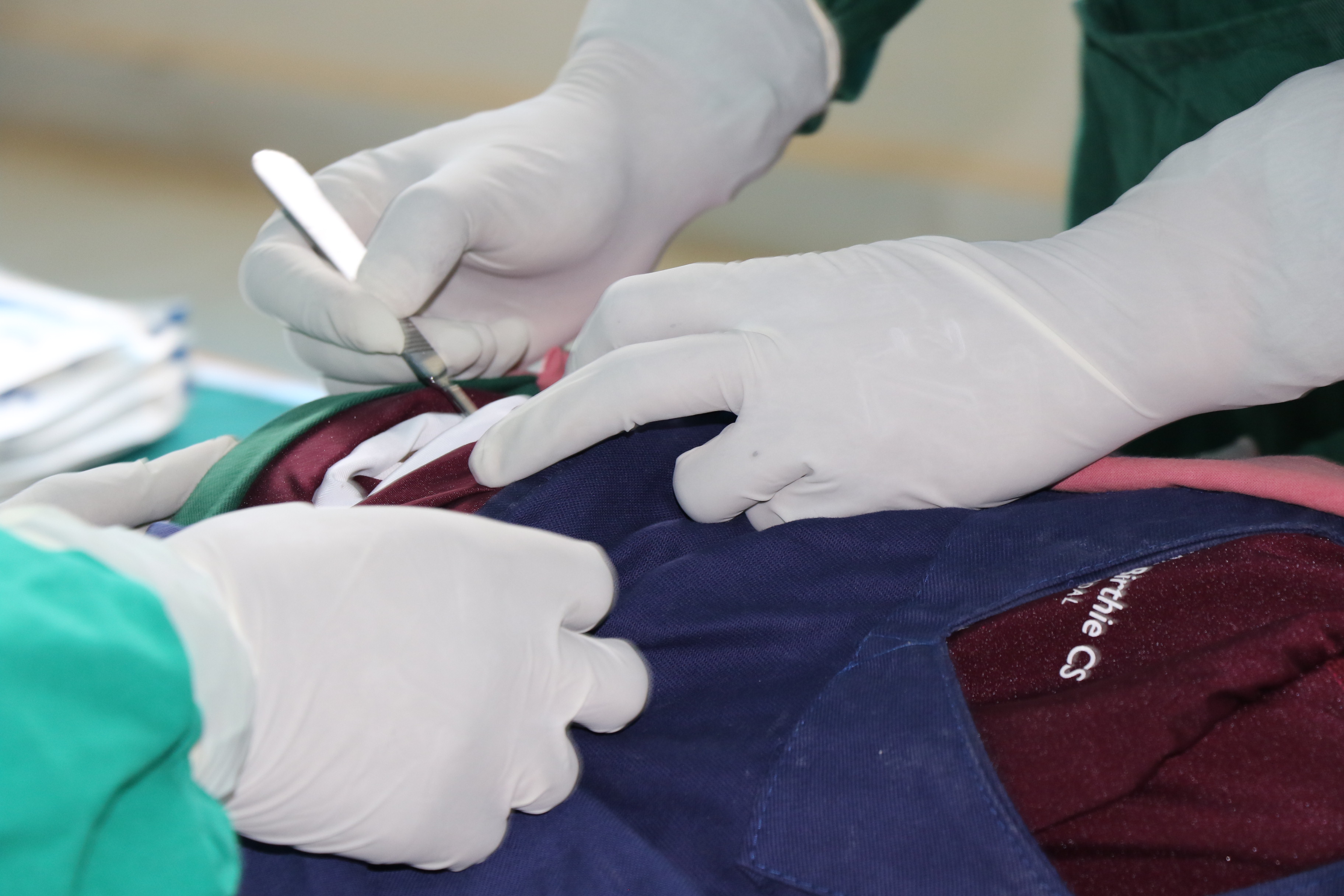


**Supplementary Figure 1:** Students practicing C-section on Mamabirthie simulator

Supplement: Supplementary file 1 — Supplementary Material 1: Figure 1. Students practicing C-section on Mamabirthie simulator [file 12909_2023_4968_MOESM1_ESM.docx]

**
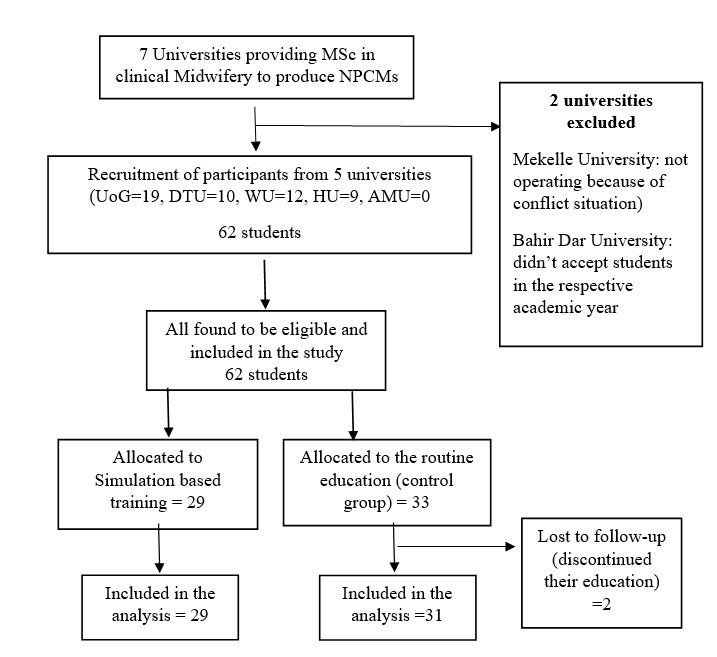
**

**Supplementary Figure 2:**Study participant selection process flow diagram

Supplement: Supplementary file 2 — Supplementary Material 2: Figure 2. Study participant selection process flow diagram [file 12909_2023_4968_MOESM2_ESM.docx]
