# Supplementary material for: Effectiveness of simulation-based cesarean section education on improving non-physician clinician midwife’s competency in performing cesarean section in Ethiopia: a quasi-experimental study
Source: BMC Med Educ. 2023 Dec 14;23:961. doi: 10.1186/s12909-023-04968-w (PMC10722683; doi:10.1186/s12909-023-04968-w)
Supplement: Supplementary file 3 — Supplementary Material 3: Table 1. Participant characteristics for simulation-based education to improve non-physician clinician midwives’ cesarean section competence in Ethiopia, 2023 [file 12909_2023_4968_MOESM3_ESM.docx]

|  | Intervention | % | Control | % | P value |
| --- | --- | --- | --- | --- | --- |
| Age | 27.2(±2.0) (24-31) |  | 28.8(±2.3) (24-34) |  | 0.006 |
| Sex |  |  |  |  |  |
| Male | 17 | 58.6 | 22 | 71 | 0.32 |
| Female | 12 | 41.4 | 9 | 29 |  |
| Workplace |  |  |  |  |  |
| Health facility | 9 | 31.0 | 10 | 32.3 | 0.92 |
| Higher education institution | 20 | 69.0 | 21 | 67.7 |  |
| Clinical experience |  |  |  |  |  |
| No clinical experience | 9 | 31.0 | 9 | 29.0 | 0.54 |
| 1-4 years | 15 | 51.7 | 13 | 42.0 |  |
| ≥5years | 5 | 17.3 | 9 | 29.0 |  |
| Marital status |  |  |  |  |  |
| Single | 16 | 55.2 | 18 | 58.1 | 0.82 |
| Married | 13 | 44.8 | 13 | 41.9 |  |
| Observed CS |  |  |  |  |  |
| Yes | 21 | 75.0 | 25 | 80.6 | 0.65 |
| No | 7 | 25.0 | 6 | 19.4 |  |
| Ever assisted CS |  |  |  |  |  |
| Yes | 7 | 24.1 | 8 | 25.8 | 0.88 |
| No | 22 | 75.9 | 23 | 74.2 |  |
| Experience in simulation-based learning |  |  |  |  |  |
| Yes | 5 | 17.2 | 10 | 32.3 | 0.18 |
| No | 24 | 82.8 | 21 | 67.7 |  |

**Supplementary Table 1:** Participant characteristics for simulation-based education to improve non-physician clinician midwives’ cesarean section competence in Ethiopia, 2023
