# Supplementary material for: Effectiveness of simulation-based cesarean section education on improving non-physician clinician midwife’s competency in performing cesarean section in Ethiopia: a quasi-experimental study
Source: BMC Med Educ. 2023 Dec 14;23:961. doi: 10.1186/s12909-023-04968-w (PMC10722683; doi:10.1186/s12909-023-04968-w)
Supplement: Supplementary file 4 — Supplementary Material 4: Table 2. Demographics characteristics of qualitative participants for simulation-based education to improve non-physician clinician midwives’ cesarean section competence in Ethiopia, 2023 [file 12909_2023_4968_MOESM4_ESM.docx]

| Participant | Age (year) | Experience (year) | Gender | Time | Category |
| --- | --- | --- | --- | --- | --- |
| P1 | 26 | 3 | Male | 30:32 | MSc clinical midwife student |
| P2 | 32 | 9 | Male | 37:14 | Gynecologist and obstetrician |
| P3 | 27 | 7 | Female | 28:12 | MSc clinical midwife student |
| P4 | 24 | 1 | Female | 22:49 | MSc clinical midwife student |
| P5 | 35 | 8 | Male | 29:25 | Gynecologist and obstetrician |
| P6 | 30 | 7 | Male | 20:09 | MSc clinical midwife student |
| P7 | 28 | 5 | Male | 17:53 | MSc clinical midwife student |
| P8 | 43 | 8 | Male | 16:11 | Gynecologist and obstetrician |
| P9 | 34 | 7 | Male | 19:32 | MSc clinical midwife student |
| P10 | 29 | 4 | Female | 16:27 | Head of the department |
| P11 | 25 | 1 | Female | 30:11 | MSc clinical midwife student |
| P12 | 25 | 2 | Male | 28:12 | MSc clinical midwife student |
| P13 | 33 | 9 | Male | 25:48 | Head of a department |
| P14 | 34 | 13 | Male | 26:20 | Head of the department |

**Supplementary Table 2:** Demographics characteristics of qualitative participants for simulation-based education to improve non-physician clinician midwives’ cesarean section competence in Ethiopia, 2023
